# Spanish-Dementia Knowledge Assessment Scale (DKAS-S): Ecuadorian validation and comparison among Spanish health students

**DOI:** 10.1186/s12877-023-03904-3

**Published:** 2023-04-01

**Authors:** Carnes-Vendrell A., Barallat-Gimeno E., Lara B., Lladó A., Escobar-Bravo MA., Reivan-Ortiz GG., Maxi-Maxi EA., Martínez-Suárez PC., Ramírez-Coronel AA., Piñol-Ripoll G.

**Affiliations:** 1 Clinical Neuroscience Research, Unitat Trastorns Cognitius, Santa Maria University Hospital, IRBLleida, Lleida, Spain; 2 Faculty of Nursing and Phisiotherapy, Universitat de Lleida, IRBLleida, Lleida, Spain; 3 Alzheimer’s Disease and Other Cognitive Disorders Unit, Department of Neurology, Hospital Clínic, Institut d’Investigació Biomèdica August Pi I Sunyer, Barcelona, Spain; 4 Laboratory of Basic Psychology, Behavioural Analysis and Programmatic Development PAD-LAB, Catholic University of Cuenca, Cuenca, Ecuador; 5 PAD-Group, Catholic University of Cuenca, Cuenca, Ecuador; 6 Laboratory of Psychometry, Comparative Psychology and Ethology, Catholic University of Cuenca, Cuenca, Ecuador; 7 Health and Behaviour Research Group (HBR), Catholic University of Cuenca, Cuenca, Ecuador; 8 Cognitive Disorders Unit, Hospital Universitari Santa Maria, Rovira Roure N° 44. 25198, Lleida, Spain

**Keywords:** Alzheimer’s disease, Dementia, Ecuador, Knowledge, DKAS, Spain, Validation studies, Students

## Abstract

**Introduction:**

Alzheimer’s disease (AD) is the most frequent cause of cognitive impairment. Improving knowledge of dementia management through health education for health professionals can improve clinical and community care in home and specialist settings. It is important to guarantee good dementia knowledge in health students, and it is necessary to evaluate it with a good standardized tool. The aim of the current study was to assess the psychometric properties of the DKAS-S with cohorts of Ecuadorian health students, to compare these results with a former validation in Spanish health students and to analyse the level of knowledge according to different variables.

**Methods:**

We performed a cross-sectional study to assess the validity, reliability and feasibility of the DKAS-S by comparing two different cohorts of health students (nursing and psychologists).

**Results:**

A total of 659 students from Spain (*n* = 233) and Ecuador (*n* = 426) completed the DKAS-S (mean age 24.02 (6.35) years old), and 52.80% were nursing students. The DKAS-S showed good internal consistency in the Ecuadorian cohort (Cronbach’s α = 0.76). No significant difference was found between Spanish and Ecuadorian students (*p* = 0.767) in the global scale score, but there were differences in some subscales. Psychologist students scored significantly higher on the global scale than nursing students (32.08 (9.51) vs. 27.49 (7.15); *p* < 0.001)). Students with a family history of cognitive impairment scored higher on the global scale, and those who had contact with people with dementia obtained better results on the global scale.

**Conclusions:**

We confirmed that the DKAS-S is an adequate and useful instrument to measure levels of knowledge about dementia among health students in Spanish-speaking communities. It is a reliable and valid measure with good psychometric properties. Understanding health students’ knowledge about dementia will allow better adaptation of academic plans to train better health professionals.

## Introduction

Alzheimer’s disease (AD) is the most frequent cause of cognitive impairment in subjects older than 65 years, representing between 60–70% of patients with cognitive impairment [1]. The global prevalence of dementia is increasing, and it will represent an economic, social, and health problem of great magnitude in the near future. According to the World Health Organization, there are 2.3 (31%) new cases per year in Europe, in contrast with the 1.2 (16%) new cases in the Americas [1]. Specifically, the global prevalence of dementia in the older population of Latin America is 11%, with higher rates among females and in urban populations [2]. The country with the highest prevalence is Colombia, with 39.30% [3], followed by Brazil, with 16.9% [4]. In contrast, Cuba is the country with the lowest prevalence of dementia, at 8.2%[5]. In Ecuador, the prevalence of dementia varies between different provinces. In Pichincha, the prevalence is 36.3% [6], and in Cumbayá Quito, it is 18–21% at 65 years old and increases to 54–60% at 85 years old [7]. The numbers confirm that Latin American and Caribbean low- and middle-income countries are at high risk. Therefore, health policies should focus on investigating not only effective pharmacological treatments but also preventive measures and ways we can improve patients’ and caregivers’ quality of life. Detecting AD early, could contribute to this improvement in quality of life [8].

Improving knowledge of dementia management through health education for health professionals can improve clinical and community care in home and specialist settings. This knowledge should start in university curricula improving recognition of the illness. Future health professionals, whether in the field of medicine, nursing, or psychology, must have this knowledge to subsequently transmit it to the general population, caregivers, and family members. Then, information and training plans can be developed for the community to improve early detection of dementia [9] and help to reduce stigma and eliminate social stereotypes [10, 11]. Ultimately, health students will be responsible for transmitting this knowledge to the general population.

To have a scale translated and validated into Spanish to assess knowledge about dementia and Alzheimer’s disease, we recently published the Spanish-Dementia Knowledge Assessment Scale (DKAS-S) [12], which has been shown to have good psychometric properties. Only three scales published before the DKAS had acceptable reliability and validity: the *Dementia Quiz* (DQ) [13], the *Knowledge of Aging and Memory Loss and Care* (KAML-C) [14] and the *Alzheimer’s Disease Knowledge Scale* (ADKS) [15].

All the above mentioned scales had some limitations such as limited scope, not updated and not covering different conceptual domains according to a systematic review that was published on 2013 [16]. Annear and collaborators found that the DKAS had superior internal consistency, a wider response distribution, a lower ceiling effect and better sensitivity to change compared with the ADKS [17].

In a previous study, we validated the DKAS-S in different cohorts from Spain, including health students. However, it is important to extend the validation in this population with a larger sample to ensure that they receive important knowledge about dementias from universities. Furthermore, to verify that the Spanish validation maintains the psychometric properties in other Spanish-speaking countries, it is necessary to replicate the study in another Spanish-speaking country by measuring its psychometric properties.

The aim of the current study was i) to assess the psychometric properties of the Spanish Dementia Knowledge Assessment Scale (DKAS-S) with cohorts of Ecuadorian health students, ii) to compare these results with the former validation in Spanish health students, and iii) to compare the level of knowledge of the disease in the two cohorts according to the different careers, gender, the presence of a family history of cognitive impairment, and having previous contact with people suffering from dementia.

## Methods

### Design

A cross-sectional study was designed to assess the validity, reliability, and feasibility of the DKAS-S.

### Sample and administration

The DKAS-S was used to test its validation and psychometric properties. From May to September 2019, we administered the scale with a cohort of health students (*n* = 233; nursing (*n* = 135) and psychologists (*n* = 98)) from the University of Lleida and Universitat Oberta de Catalunya in Spain. Afterwards, from June to December 2021, it was also administered to a cohort of health students (*n* = 426; nursing (*n* = 213) and psychologists (*n* = 213)) from Catholic University of Cuenca in Ecuador. The fact that the scale was administered in two periods did not affect the results and it is a consequence of the need of validating first the scale into the Spanish population before doing any further validation with other populations.

The DKAS-S comprises 25 statements about dementias, and subjects are asked to answer on a Likert scale with five response options: true, probably true, probably false, false, and don’t know. It has good psychometric properties with Cronbach’s alpha coefficient of 0.819 while the scores for each of the subscales were lower and ranged from 0.556 to 0.718 [12].

We obtained written informed consent from all the participants before including them. Participation was completely voluntary. All participants followed the same procedure to complete the data collection sheet. After signing the informed consent form, they were given a copy of the scale, which they completed in approximately 15 min. Participant anonymity and confidentiality were guaranteed. The Scientific Ethics Committee of the Hospital Universitari Arnau de Vilanova approved both the study and the consent procedure (CEIC 2119), as well as the Committee of Bioethics in Research of the Health Area (COBIAS) from the Catholic University of Cuenca in Ecuador (2022–011 EOIE).

### Statistical analysis

Statistical analyses were performed following the steps of the construction of the original scale [15] and from the Spanish adaptation [12]. First, a descriptive analysis of all the variables in the sample for each group (Spanish and Ecuadorian students) was performed by measuring the central tendency and dispersion of quantitative data and the frequency distribution of qualitative data. Second, the means of the responses in each group to the final scale were compared using ANOVA. Third, psychometric analyses of the scale were performed for the Ecuadorian cohort. Internal consistency analyses of the full scale and each of the subscales that compose it were made using Cronbach’s alpha. Additionally, the validity of the construct was verified by exploratory factor analysis of principal components with varimax rotation and a fixed number of 4 factors (the ones that had the original scale). Finally, bivariate analysis was performed using Student’s t test for quantitative variables. All statistical analyses were performed using SPSS 24.0 (SPSS, Chicago, IL), and the level of significance was set at 0.05.

## Results

### Participant characteristics

In total, 659 students from Spain and Ecuador completed the DKAS-S (Table 1), of which 52.8% (*n* = 348) were nursing students. All of the students were in the first or second year of their studies and none of them had previous training or knowledge from another degree. The mean age was 24.02 (6.35) years. Only 24.1% had been in contact with people with dementia, and 22% had a family history of cognitive impairment. Only 38.2% of the Spanish students were working during their studies while none of the Ecuadorian students were working at the time of the administration of the scale. All the characteristics of the population are shown in Table 1.

**Table 1:** Demographic characteristics of Spanish-Dementia Knowledge Assessment Scale (DKAS-S) responders in Spanish and Ecuatorian students

	**Total**	**Spanish students**	**Ecuatorian students**
	***n* = 659**	***n* = 233**	***n* = 426**
**Female (%)**	75.9%	82.8%	72.1%
**Mean age (SD)**	24.04 (6.35)	26.3 (9.2)	22.8 (3.33)
**Contact with dementia people**			
**No**	75.9%	50.4%	89.7%
**Yes**	24.1%	49.6%	10.3%
**Family history of cognitive impairment**			
**Yes**	22%	41.2%	11.5%
**Career**			
**Nursing students**	348	135	213
**Psychologist students**	311	98	213

### Internal consistency of the Ecuador cohort

The DKAS-S had good internal consistency, with a Cronbach’s alpha coefficient of 0.76 exceeding the acceptability criterion of > 0.70, and was consistent with those of other validated scales reported in the health literature [18]. However, the internal consistency of each subscale was not that good: 0.65 causes and characteristics, 0.53 communication and behaviour, 0.56 care considerations and 0.38 health and risk promotion.

### Exploratory factor analysis of the Ecuador cohort

The validity of the construct was verified by confirmatory factor analysis. For most of the items, the eigenvalue was good, approaching the acceptability criterion of > 0.20 (Table 2). However, there were challenges in the inclusion of some items in the original subscale. These items were 2 and 6 from subscale 1 (causes and characteristics), item 14, 17 and 18 from subscale 2 (communication and behaviour), item 20 from subscale 3 (care considerations), and items 9, 12, and 13 from subscale 4 (health risk and promotion).

**Table 2:** Pattern matrix for the 25-item Spanish-Dementia Knowledge Assessment Scale (DKAS-S) in the Ecuador cohort

	**Subscale 1: causes and characteristics**	**Subscale 2: health risk and promotion**	**Subscale 3: communication and behaviour**	**Subscale 4: care considerations**
Item 1	0.612			
Item 2				0.614
Item 3	0.514			
Item 4	0.622			
Item 5	0.708			
Item 6		0.541		0.289
Item 7	0.456	0.328		
Item 8				0.657
Item 9	0.458	0.257		
Item 10				0.606
Item 11			0.387	0.312
Item 12	0.615			
Item 13		0.712		
Item 14	0.372	0.403		
Item 15		0.726		
Item 16		0.500		
Item 17			0.259	0.479
Item 18	-0.312	0.298		0.421
Item 19		0.624		
Item 20	0.696			
Item 21			0.521	0.322
Item 22			0.680	
Item 23			0.728	
Item 24			0.652	
Item 25			0.557	

### Discrimination between cohorts

**Fig. 1:**
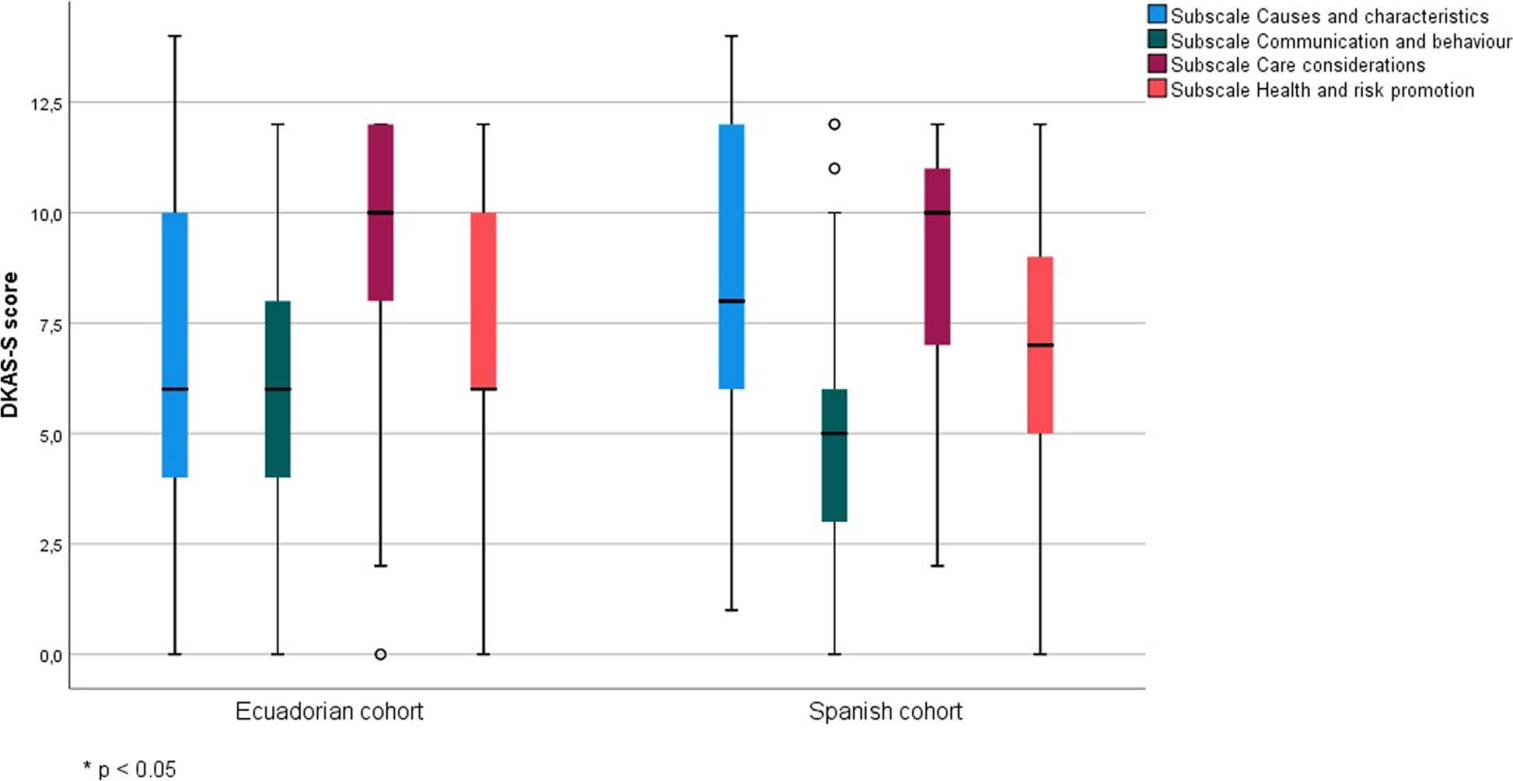
Comparison of DKAS-S scores subescales in Spanish and Ecuadorian students

The mean score for all subjects on the scale was 30.17 (8.75) points of a total possible score of 50. No significant difference was found between Spanish and Ecuadorian students (*p* = 0.134) in the global scale score (Table 3). The DKAS-S is divided into four subscales: knowledge of causes and characteristics, communication and behaviour, care considerations, and health risk and promotion. There were statistically significant differences between the two cohorts in the causes and characteristics subscale, where Spanish students scored 8.81 (3.31) points, which was significantly higher than Ecuadorian students who scored 7.02 (3.81) points (*p* < 0.001). The Ecuadorian students scored significantly higher on the communication and behaviour subscale (7.44 (3.07 vs. 5.00 (2.33); *p* < 0.001) and the health and risk promotion subscale (7.41 (2.78) vs. 6.82 (2.45); *p* = 0.008). The results are also shown in Fig. 1.

**Table 3:** Discrimination between cohorts

	**Total**	**Spanish students**	**Ecuatorian students**	***p value***
	***n* = 659**	***n* = 233**	***n* = 426**	
**Total score**	30.17 (8.75)	29.59 (7.65)	30.53 (9.29)	0.134
**Score Causes and characteristics**	7.65 (3.74)	8.81 (3.31)	7.02 (3.81)	< 0.001***
**Score Health risk and promotion**	7.20 (2.69)	6.82 (2.45)	7.41 (2.78)	0.008*
**Score Communication and Behaviour**	6.57 (3.06)	5.00 (2.33)	7.44 (3.07)	< 0.001***
**Score Care considerations**	8.73 (2.73)	8.87 (2.59)	8.65 (2.80)	0.343

Level of statistical significance = *p < 0.05, **p < 0.01, ***p < 0.001

**Fig. 2:**
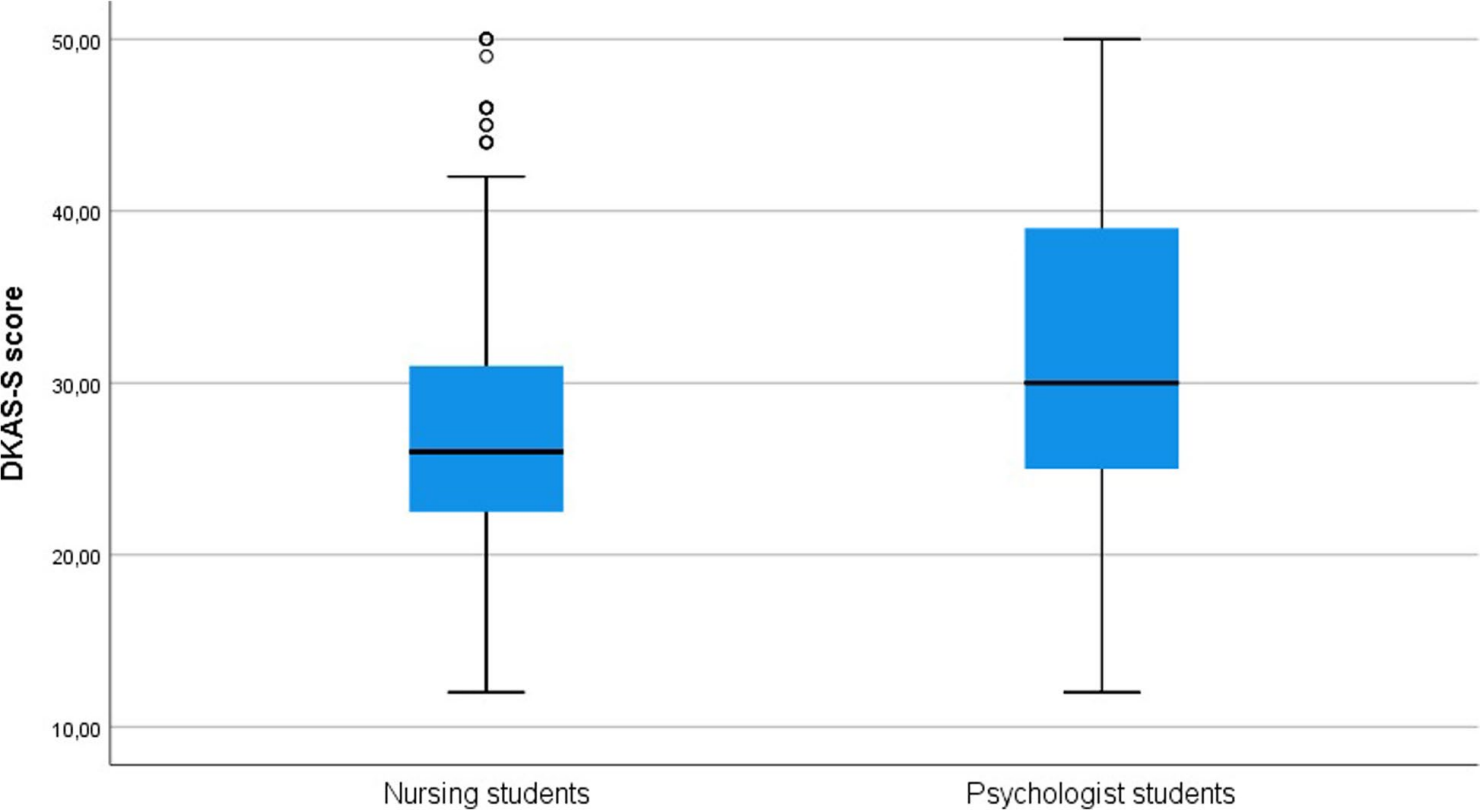
Comparison of DKAS-S scores between psychology and nursing students

Discrimination between careers, gender, family history of cognitive impairment and, contact with people suffering from dementia:

Psychology students scored significantly higher than nursing students (31.32 (8.77) vs. 29.14 (8.62); *p* = 0.001)) (Fig. 2). In addition, the differences remain statistically significant in all the subscales except for care considerations. No significant difference was found between males and females (*p* = 0.239) in the global scale score. However, there were differences in the causes and characteristics subscale, where females scored higher (7.83 (3.67)) than males (7.11 (3.93)) (*p* = 0.036). Students with a family history of cognitive impairment scored higher on the global scale (*p* = 0.002) and on the causes and characteristics (*p* < 0.001) and care considerations subscales (*p* = 0.002)). Finally, students who had contact with people with dementia obtained better results on the global scale (*p* = 0.015) and on the causes and characteristics (*p* < 0.001) and care considerations subscales (*p* = 0.017)). See all the results in Table 4.

**Table 4:** Discrimination between careers, gender, family history of cognitive impairment and contact with dementia people

	**Nursing students**	**Psychologist students**	***p value***	**Female**	**Male**	***p value***	**Yes**	**No**	***p value***	**Yes**	**No**	***p value***
	***n* = 348**	***n* = 311**										
**Total score**	29.14 (8.62)	31.32 (8.77)	0.001**	30.40 (8.54)	29.46 (9.39)	0.239	32.12 (8.63)	29.62 (8.72)	0.002*	31.66 (8.22)	29.72 (8.87)	0.015*
**Score Causes and characteristics**	7.34 (3.25)	8.00 (4.21)	0.027*	7.83 (3.67)	7.11 (3.93)	0.036*	8.89 (3.66)	7.30 (3.69)	< 0.001***	8.84 (3.70)	7.28 (3.68)	< 0.001***
**Score Health risk and promotion**	6.81 (2.67)	7.64 (2.64)	< 0.001***	7.15 (2.67)	7.37 (2.72)	0.376	7.36 (2.70)	7.16 (2.68)	0.462	7.24 (2.49)	7.19 (2.75)	0.823
**Score Communication and Behaviour**	6.35 (3.17)	6.83 (2.92)	0.045*	6.58 (2.99)	6.55 (3.28)	0.902	6.51 (3.12)	6.59 (3.05)	0.782	6.38 (3.01)	6.65 (3.08)	0.338
**Score Care considerations**	8.63 (2.75)	8.84 (2,71)	0.338	8.83 (2.67)	8.42 (2.91)	0.104	9.35 (2.54)	8.55 (2.76)	0.002*	9.18 (2.55)	8.59 (2.77)	0.017*

Level of statistical significance = *p < 0.05, **p < 0.01, ***p < 0.001

## Discussion

The research demonstrated that the DKAS-S showed good psychometric properties for validity, reliability, and factorial analysis in an Ecuadorian health student population. The DKAS-S in Ecuador had good internal consistency, which indicates that all the items measured the same underlying construct of dementia knowledge. Although the internal consistency in the Ecuador cohort was lower than that in the Spanish cohort, it still proved that it is a good scale to measure the level of dementia knowledge.

Compared with previous validations, the DKAS-S internal consistency still exceeds the Japanese validation [19] but not the traditional Chinese validation [20], which obtained a Cronbach's alpha of 0.93. However, the Chinese validation only includes home care workers and did not consider health students, as this study and the Japanese study did. Comparing the DKAS-S with the only other scale validated in Spanish, the DKAT2 [21], both have the same internal consistency, and both were validated with nursing students.

Both Spanish and Ecuadorian students had good knowledge about dementia, with a global score of 29.65 out of 50 points (59.3%) and with no significant differences between the two countries. However, we did find significant differences in some subscales. The Spanish students showed better knowledge about the causes and characteristics of dementias and AD, whereas Ecuadorian students knew more about communication and behaviour. These differences may be due to differences in curricula between the two countries and/or cultural differences. Latin Americans, and therefore Ecuadorians, tend to be more focused on attention to and care of the elderly. The importance of family social capital in the intergenerational reciprocity among mothers, daughters, fathers, and sons in Ecuador has been demonstrated in recent qualitative studies [22], which would explain their greater knowledge in the communication and behaviour subscale. Instead, in a study carried out in Spain, although intergenerational family solidarity continues to exist, the structure and family dynamics have changed. The high rates of an active female population that Spain has recently reached affect families’ preferences and care strategies [23]. While in Ecuador the value of the family is a social capital, in Spain, policies are being initiated to promote more open, flexible, and accessible labour markets that allow reconciling professional life and family life. In Spain, the percentage of men and women with higher education is above the EU average (33.0% for men, 38.4% for women) [23]. This distinction could explain the better understanding of the clinical disease by Spanish students.

Compared with previous studies, the knowledge of dementia in the student population is approximately 60–65% of the maximum score. A study of the DKAT2-Sp with nursing students obtained 61.2% of the maximum score [21], and using the ADKS in Malta, nursing students had a knowledge of dementia of 64.5% of the maximum score [24]. In another study, Eccleston et al. (2015) evaluated knowledge of dementia (using the DKAT2) before and after nursing students’ participation in supported clinical placements at an interventional residential care facility [25]. The results showed that the level of dementia knowledge at baseline was poor but significantly improved after students’ participation in the intervention compared to those who had only attended clinical placements at control facilities. Therefore, in general, all the scales that measure the level of dementia knowledge in nursing students achieve the same percentage. In our sample, we included psychology students because psychologists also contribute to the diagnosis and treatment of dementias and helping caregivers. In fact, we find that psychologists had more knowledge of dementia (64,16%) than nursing students (54,98%), which was statistically significant. This difference could be due to the curricula of both careers. Nursing studies are more focused on practical aspects related to the handling of medical instruments and nursing care, whereas psychology studies are more focused on knowing the theoretical aspects of different mental illnesses (including dementias) and their therapeutic management.

Another important aspect to note about the DKAS-S (and other scales) is that it has an “I don’t know” option, which is important because it allows respondents to declare their ignorance of a topic, without having to say “Yes” or “No”. This option also avoids the bias of correct responses at random. Previous studies have demonstrated the benefits of including an “I don’t know” option in questionnaires [26, 27]. Specifically, Dolnicar and Rossiter (2009) indicate that data contamination can be quite substantial in cases where a “Don’t know” option is not offered and respondents are asked to make statements about a topic with which they are not familiar or about which they do not know enough [27]. In addition, this option allows us to identify topics on which there is poor knowledge. Without going into detail about what these answers have been for obvious reasons of space, at a glance we have been able to detect that the percentage of answers "I don't know" of the sample was 4.06% If we analyse further these answers and add the incorrect responses (erroneous knowledge), we can obtain the aspects in which it is necessary to improve training programmes. This step is necessary to improve the training of future health professionals. The more prepared they are, the better they can meet the demands of patients and their caregivers. Tools such as DKAS-S are needed to measure the level of knowledge they receive from universities.

For our third aim, we compared the level of knowledge based on different variables. As expected, students with a family history of cognitive impairment had more knowledge about dementia, as did students who had contact with people with dementia. The same results were found in the DKAT2-Sp validation, where nursing students with experience in caring for family members with dementia scored better on the DKAT2-Sp scale (which was also statistically significant compared with those who had not) [21]. In contrast, Scerri and Scerri (2013) did not find significant differences in the ADKS score comparing students who had family members with dementia [24]. However, they did find differences in nursing students who had a history of caring for persons with dementia during clinical placement who had better knowledge. Despite being students and being in the learning process, having been in contact with people with dementia, either in their own family or through acquaintances, made them more knowledgeable than their peers. Instead, we did not find statistically significant differences in the level of knowledge of dementias by gender. Both males and females had the same knowledge.

Our research has some limitations. We could not analyse the level of knowledge by the students’ academic year. This analysis would have allowed us to know when the greatest learning occurs and to correlate it with the study plans to see where they can be improved. In addition, we did not include medical students, which would be interesting because they are also an important part of the health system. However, our investigation also has some strengths. It is based on a large sample, including not only nursing students similar to most previous studies but also psychologists. Another added strength is the replication of the validation of the DKAS-S in another Spanish-speaking population in Latin America. This finding guarantees that the scale adequately represents the contents that are intended to be evaluated (content validity).

## Conclusions

In conclusion, we have confirmed that the DKAS-S is an adequate and useful instrument to measure levels of knowledge about dementia, in this case among health students in Spanish-speaking communities. It is a reliable and valid measure with good psychometric properties. Understanding health students’ knowledge of dementia will allow better adaptation of academic plans to train better health professionals.

## Data Availability

The datasets used and analysed during the current study available from the corresponding author on reasonable request.

## Abbreviations:

AD: Alzheimer’s disease
DKAS-S: Spanish dementia knowledge assessment scale
